# Complete Genome sequence of *Burkholderia phymatum* STM815^T^, a broad host range and efficient nitrogen-fixing symbiont of *Mimosa* species

**DOI:** 10.4056/sigs.4861021

**Published:** 2014-03-25

**Authors:** Lionel Moulin, Agnieszka Klonowska, Bournaud Caroline, Kristina Booth, Jan A.C. Vriezen, Rémy Melkonian, Euan K. James, J. Peter W. Young, Gilles Bena, Loren Hauser, Miriam Land, Nikos Kyrpides, David Bruce, Patrick Chain, Alex Copeland, Sam Pitluck, Tanja Woyke, Michelle Lizotte-Waniewski, Jim Bristow, Margaret Riley

**Affiliations:** 1 IRD, UMR-LSTM, Campus de Baillarguet 34398 Montpellier cedex 5; France; 2 Univ Massachussets, MA, USA; 3 James Hutton Institute, Dundee, United Kingdom; 4 University of York, United Kingdom; 5 Oak Ridge National Laboratory, Oak Ridge, TN, USA; 6 Joint Genome Institute, Walnut Creek, CA, USA; 7 Los Alamos National Laboratory, Los Alamos, NM, USA

**Keywords:** *Burkholderia*, symbiosis, *Mimosa*, rhizobia, nitrogen fixation

## Abstract

*Burkholderia phymatum* is a soil bacterium able to develop a nitrogen-fixing symbiosis with species of the legume genus *Mimosa*, and is frequently found associated specifically with *Mimosa pudica*. The type strain of the species, STM 815^T^, was isolated from a root nodule in French Guiana in 2000. The strain is an aerobic, motile, non-spore forming, Gram-negative rod, and is a highly competitive strain for nodulation compared to other *Mimosa* symbionts, as it also nodulates a broad range of other legume genera and species. The 8,676,562 bp genome is composed of two chromosomes (3,479,187 and 2,697,374 bp), a megaplasmid (1,904,893 bp) and a plasmid hosting the symbiotic functions (595,108 bp).

## Introduction

Rhizobia are a functional class of bacteria able to enter into nitrogen-fixing symbioses with legumes. The bacterial symbiont induces the formation of nodules on the roots of the plant where they differentiate into nitrogen-fixing bacteroids. *Bacteria* then allocate combined nitrogen to the plant, which in return provides the bacteria with energy derived from photosynthesis. This symbiosis confers agricultural advantages to the legumes by reducing the need for fertilization and allows them to be pioneer plants on degraded or contaminated soils.

Rhizobia are polyphyletic and are placed within two classes of *Proteobacteria*, the *Alphaproteobacteria* and the *Betaproteobacteria*. They are closely related to non-symbiotic species, including important human, animal or plant pathogens or saprophytes. Most research has focused on the α-rhizobia, since the β-rhizobia were only recently discovered [1,2]. The α-rhizobia include 10 genera (*Sinorhizobium*, *Mesorhizobium*, *Rhizobium*, *Methylobacterium*, *Devosia*, *Azorhizobium*, *Bradyrhizobium*, *Ochrobactrum*, *Bosea* and *Phyllobacterium*) and have a worldwide distribution associated with a diversity of legume species (from herbs to trees). To date, the β-rhizobia include only two genera, *Burkholderia* and *Cupriavidus* (ex *Ralstonia*), and a dozen species (for review [3], updated in [4]). They are found preferentially associated with *Mimosa* species (at least 68 nodulated species, and especially *M. pudica*, *M. pigra*, and *M. bimucronata*) in Asia, Australia, and Central and South America [5,6]. Based on a comparison of house-keeping and nodulation gene phylogenies, *Burkholderia* species have been postulated to be ancestral symbionts of South American *Mimosa* and *Piptadenia* species [4,5]. Here we describe the genome sequence of one of the first described β-rhizobia, the type strain of *Burkholderia phymatum*, STM815^T^.

## Classification and features

**Figure 1:**
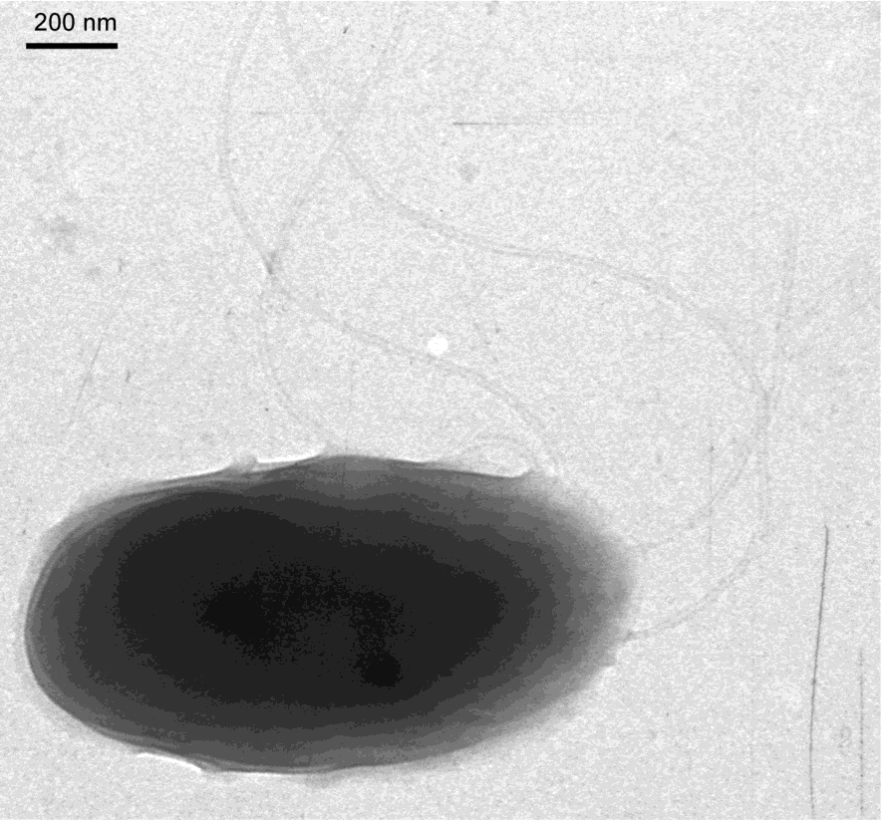
Transmission electron microscopy of *B. phymatum* STM815 (credit: Geoffrey Elliott).

**Figure 2:**
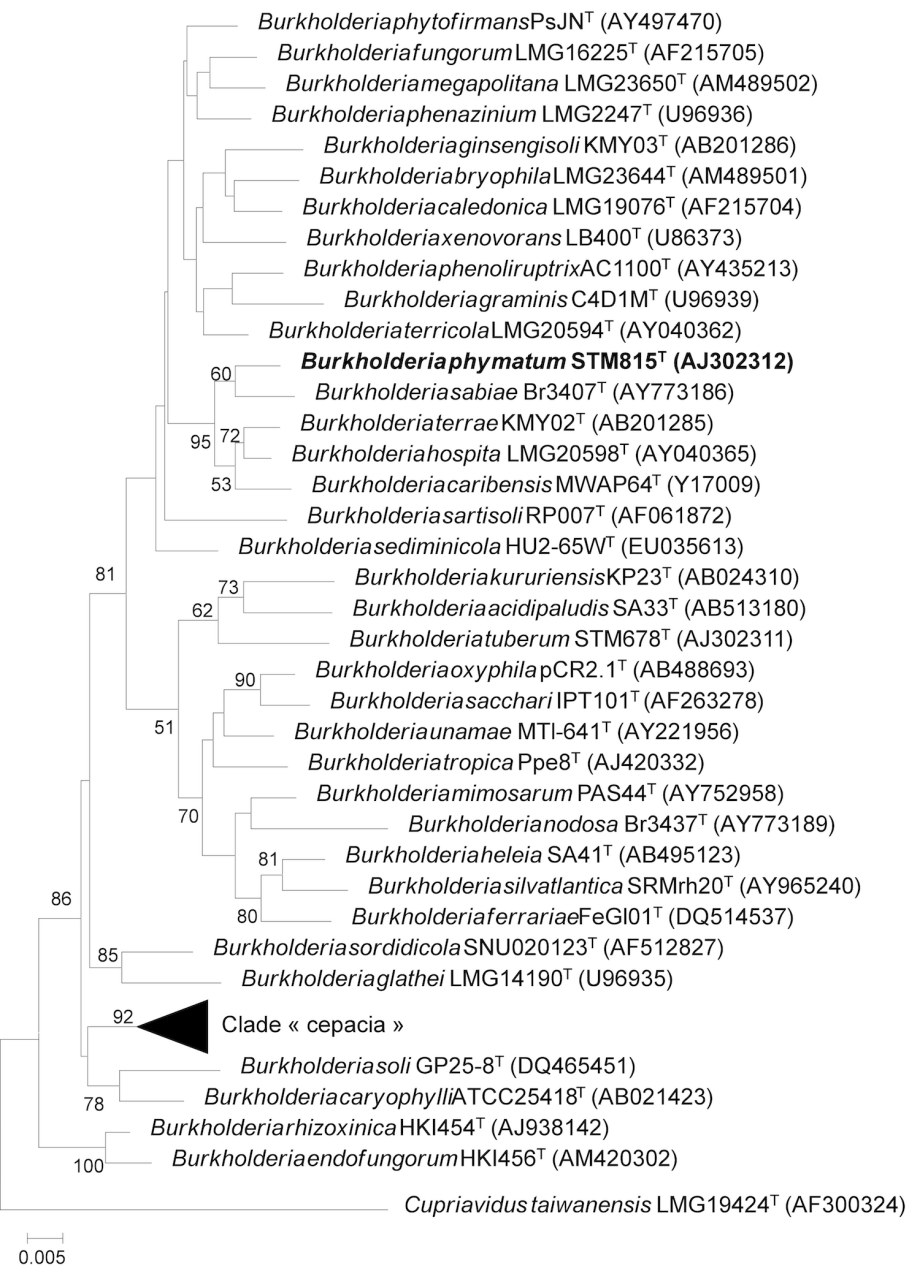
Phylogenetic tree highlighting the position of *Burkholderia phymatum* strain STM815^T^ relative to other type strains within the genus *Burkholderia*. The 16S rDNA sequences from type strains were obtained from the ribosomal database project [13], aligned with muscle 3.6, and a neighbor-joining tree was built from a Kimura-2P corrected distance matrix using BioNJ on the www.phylogeny.fr server [14]. Numbers at nodes are % bootstraps from 1000 replicates (shown only if >50%). Accession numbers of 16S rDNA are indicated between parentheses for each strain. *C. taiwanensis* LMG19424^T^ was used as outgroup.

*Burkholderia phymatum* STM815^T^ is a motile, Gram-negative rod (Figure 1) in the order *Burkholderiales* of the class *Betaproteobacteria*. It is fast growing, forming colonies within 3-4 days when grown on yeast-mannitol agar (YMA [7],) at 28°C. It is one of the first described members of the β-rhizobia. The strain STM815^T^, which is the type strain of the species, was isolated from nodules of *Machaerium lunatum* in French Guiana in 2000 [1], and the species, *B. phymatum,* was described based on this single isolate [8]. However, the species has subsequently been shown not to nodulate *Machaerium* species [9], but it can nodulate species in the large genus *Mimosa* [9,10]. Indeed, the symbiotic abilities of STM815^T^ have been demonstrated on numerous *Mimosa* species, and this strain is now considered to be an efficient symbiont of a broad range of legumes, particularly in *Mimosa* and related genera in the sub-family *Mimosoideae* [9]. Strain STM815^T^ is also able to fix nitrogen in free-living conditions [9]. Many isolates of *B. phymatum* have been sampled from *Mimosa pudica* in French Guiana [10], Papua New Guinea [9], China [11] and India [12]. Phylogenetic analyses of core and symbiotic genes have illustrated the ancestral status of *Burkholderia* species in symbioses with *Mimosa* [4,5]. *Burkholderia phymatum* STM815^T^ is now considered to be a model system for studying the adaptive processes of *Burkholderia* in symbioses with legumes, in comparison with α-rhizobia. The *B. phymatum* species is phylogenetically related to symbiotic and non-pathogenic species, and is distant from the “cepacia” clade of *Burkholderia* (which contains many pathogenic species) (Figure 2, Table 1).

**Table 1:** Classification and general features of *Burkholderia phymatum* STM815 according to MIGS recommendations [15]

**MIGS ID**	**Property**	**Term**	**Evidence code^a^**
		Domain *Bacteria*	TAS [16]
		Phylum *Proteobacteria*	TAS [17]
		Class *Betaproteobacteria*	TAS [18,19]
	Current classification	Order *Burkholderiales*	TAS [18,20]
		Family *Burkholderiaceae*	TAS [18,21]
		Genus *Burkholderia*	TAS [22-24]
		Species *Burkholderia phymatum*	TAS [8,25]
		Type strain STM815	
	Gram stain	negative	TAS [8]
	Cell shape	straight rods	TAS [8]
	Motility	motile	
	Sporulation	non-sporulating	TAS [8]
	Temperature range	mesophile, no growth at 42°C	TAS [8]
	Optimum temperature	28°C	TAS [8]
	Carbon source	D-glucose, L-arabinose, D-mannose, D-mannitol, N-acteyl-D-glucosamine, D-gluconate, caprate, D-galactose, citric acid, D-galacturonate acid, methyl-pyruvate, L-aspartic acid, L-glutamic acid, L-asparagine, D,L-lactic acid	TAS [8] TAS [8] IDA IDA IDA IDA
	Energy source	chemoorganotroph	TAS [8]
MIGS-6	Habitat	Soil, nodule, host	TAS [1]
MIGS-6.3	Salinity	Not reported	
MIGS-22	Oxygen	Aerobic	TAS [8]
MIGS-15	Biotic relationship	Free living, Symbiotic	TAS [1,9]
MIGS-14	Pathogenicity	None	
MIGS-4	Geographic location	Root nodule of *Machaerium lunatum* in French Guiana (Paracou)	TAS [1]
MIGS-5	Sample collection time	2000	TAS [1]
MIGS-4.1	Latitude	5°15’N	TAS [1]
MIGS-4.2	Longitude	52°55’W	TAS [1]
MIGS-4.3	Depth	Not reported	
MIGS-4.4	Altitude	32 m	TAS [1]

a) Evidence codes - IDA: Inferred from Direct Assay; TAS: Traceable Author Statement (i.e., a direct report exists in the literature); NAS: Non-traceable Author Statement (i.e., not directly observed for the living, isolated sample, but based on a generally accepted property for the species, or anecdotal evidence). These evidence codes are from the Gene Ontology project [26].

### Symbiotaxonomy

*Burkholderia phymatum* STM815^T^ forms nodules (Nod^+^) and fixes N_2_ (Fix^+^) with a broad range of *Mimosa* species [6,9] as well as with other genera in the tribe *Mimoseae* in the *Mimosoideae* legumes sub-family [9]. Nodulation data were compiled in Table 2.

**Table 2:** Mimosoid legumes tested for nodulation with *Burkholderia phymatum* STM815

Tribe / Genus	Species	Nodulation by STM815
Tribe Mimoseae*		
*Acacia*	*farnesiana, karroo, nilotica* var. *kraussiana, nilotica* var. *leiocarpa*, *pennatula, schaffneri, seyal, tortilis*	F
*Anadenanthera*	*pavonina, colubrina*	F
*Desmanthus*	*bicornutus*, *fruticosus, virgatus*	O
*Dichrostachys*	*cinerea*, *microcephala*	O
*Leucaena*	*collinsii*, *cuspidata*, *pulverulenta*, *trichodes*	N
	*confertiflora, esculenta, greggii, retusa, salvadorensis*	O
	*leucocephala, multicapitula*	F
*Microlobius*	*foetidus*	O
*Mimosa*	*aculeaticarpa*^1^, *luisana*^1^, *setosissima*^4^	O
	*acutistipula*^1^, *albida*^1^, *albolanata*^4^, *artemisiana*^1^, *bimucronata*^1^, *caesalpiniifolia*^1^, *camporum*^1^, *cordistipula*^4^, *debilis*^4^, *diplotricha*^1^, *foliolosa*^4^, *flocculosa*^1^, *hexandra*^1^, *himalayana*^1^, *invisa*^1^, *latispinosa*^1^, *ophtalmocentra*^1^, *pigra*^1^, *polydactyla*^1^, *pudica*^1^, *somnians*^1^, *tenuiflora*, *setosa*^4^, *ursina*^4^, *velloziana*^4^, *xanthocentra*^4^	F
	*adenocarpa*^1^, *affinis*^1^, *bahamensis*^1^, *blanchetii*^1^, *borealis*^1^, *callithrix*^4^, *claussenii*^4^, *decorticans*^4^, *delicatula*^1^, *densa^4^*, *dysocarpa*^1^, *melanocarpa*^4^, *menabeensis*^1^, *polyantha*^1^, *scabrella*^1^, *uruguensis*^1^	I
*Neptunia*	*dimorphantha*, *gracilis*, *major*e, *monosperma*, *plena*	O
	*oleracea*	N
*Parapiptadenia*	*rigida*	N
*Piptadenia*	*gonoacantha*, *stipulacea*, *viridiflora*^2^	F
*Pityrocarpa*^3^	*moniliformis*, *obliqua*	F
*Prosopis*	*africana*, *farcta*, *glandulosa*, *velutina*	O
	*chilensis*, *pubescens*	N
	*juliflora*	F
*Schleinitzia*	*novo-guineensis*	O
*Stryphnodendron*	*coriaceum*, *guianensis*, *pulcherrimum*	O
**Tribe *Ingeae*†**		
*Acacia* (Ac)	*senegal*	N
*Acacia* (P)	*dealbata*	O
	*mangium*	N
*Albizia*	*adenocephala, kalkora*, *niopoides*	O
	*julibrissin*	N
*Calliandra*	*houstiana* var. *acapulcens, houstiana* var. *anomala*, *houstiana* var. c*alothyrsus*, *juzepczukii*, *trinervia*	F
	*physocalyx, rubescens*	N
*Chloroleucon*	*tortum*	O
*Enterolobium*	*cyclocarpum*	O
*Faidherbia*	*albida*	N
*Pithecellobium*	*dulce*	F
*Samanea*	*saman*	O
*Zapoteca*	*tetragona*	O

Legend: O = no nodules formed; N = outgrowths on roots, superficially similar to nodules but ineffective; I = nodules formed are inefficient; F = nitrogen fixing nodules formed (these may not all be fully effective, but plants gave acetylene reduction values at least twice that of non-nodulated control plants).

*This is taken to include *Acacia* subgenus *Acacia*, now thought to be closely related to tribe Mimoseae and given the generic name *Vachellia* by some.

^†^This is taken to include *Acacia*, subgenera *Aculeiferum* (Ac) and *Phyllodineae* (P). The species listed below are now also included in genera *Senegalia* and *Acacia* respectively. Species from other genera in former *Acacia* have not been studied here.

^1^ Nodulation data from [9]; ^2^ This species has been transferred to an as yet unnamed genus by [27]; ^3^ This genus was formerly in *Piptadenia* [27]; ^4^ Nodulation data from [6]. Nodulation data for other legumes are from unpublished data from E.K. James and L. Moulin.

## Genome sequencing information

### Genome project history

The genome was selected by a consortium of researchers led by M. Riley, to be sequenced by the DOE Joint Genome Institute as part of the “Recommendations for Sequencing Targets in Support of the Science Missions of the Office of Biological and Environmental Research”. Initially, the strain was chosen to enrich genome data in the *Burkholderia* genus for comparative genomics. The genome was selected for genome determination because strain STM815^T^ is a legume symbiont, as compared to the large number of genome sequences available for opportunistic and human-pathogens. The genome sequence was completed in 2007 and presented for public access on April 2008. Automatic annotation was performed using the JGI-Oak Ridge National Laboratory annotation pipeline [28]. Additional automatic and manual sequence annotation, as well as comparative genome analysis, were performed using the MicroScope platform at Genoscope [29]. Table 3 presents the project information and its association with MIGS version 2.0 compliance [30].

**Table 3:** Project information

**MIGS ID**	**Property**	**Term**
MIGS-31	Finishing quality	Complete
MIGS-28	Libraries used	3 kb, 8 kb and 40 kb (fosmid)
MIGS-29	Sequencing platforms	Sanger
MIGS-31.2	Fold coverage	11.2
MIGS-30	Assemblers	Phred/Phrap/Consed
MIGS-32	Gene calling method	DOE-JGI tools
	Genome Database release	December 12, 2008
	Genbank ID	CP001043 - CP001046
	Genbank Date of Release	April 22, 2008
	NCBI BioProject ID	PRJNA17409
	GOLD ID	Gc00775
	Project relevance	biotechnological

### Growth conditions and DNA isolation

The strain was grown in 50 ml of broth Yeast-mannitol medium (YM [7],) and DNA isolation was performed using a CTAB (Cetyl trimethyl ammonium bromide) bacterial genomic DNA isolation method [31].

### Genome sequencing and assembly

The genome of *Burkholderia phymatum* STM815^T^ was sequenced by Sanger technology at the Joint Genome Institute (JGI) using a combination of 3 kb, 8 kb and 40 kb (fosmid) DNA libraries. All general aspects of library construction and sequencing performed at the JGI can be found at the DOE JGI website [32].

Draft assemblies were based on 115,329 total reads and resulted in approximately 11.2× coverage of the genome. The Phred/Phrap/Consed software package was used for sequence assembly and quality assessment [33-35]. Gaps between contigs were closed by custom primer walks on gap spanning clones or PCR products. A total of 1,282 additional reactions were necessary to close gaps and to raise the quality of the finished sequence. The completed genome sequences of *B. phymatum* STM815^T^ contain 115,487 reads, achieving an average of 11.2-fold sequence coverage per base with an error rate less than 1 in 100,000.

### Genome annotation

Automatic annotation was performed using the Integrated Microbial Genomes (IMG) platform [36] developed by the Joint Genome Institute, Walnut Creek, CA, USA [28]. Additional automatic and manual sequence annotation, as well as comparative genome analysis, were performed using the MicroScope platform at Genoscope [29]. Gene calling in Microscope resulted in the prediction of 940 additional protein coding sequences compared to the 7,496 detected at IMG. These additional genes were mostly short coding sequences considered as gene remnants or fragmented CDS, so that genome statistics presented here are from the IMG platform.

## Genome properties

**Figure 3:**
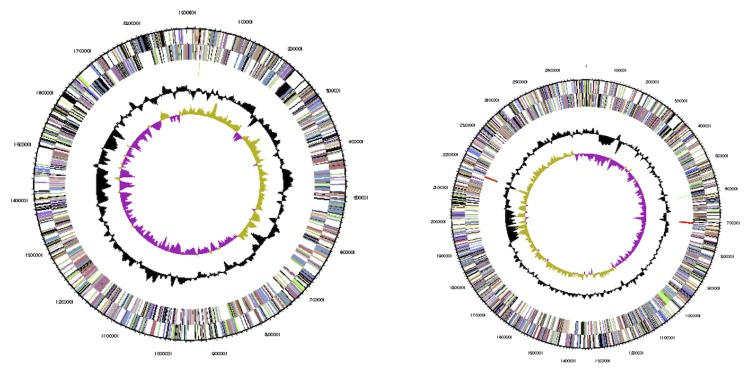
Circular maps of Chromosome 1 (left) and Chromosome 2 (right) of *B. phymatum* STM815^T^. From outside to center: Genes on forward strand (color by COG categories as denoted by the IMG platform), Genes on reverse strand (color by COG categories), RNA genes (tRNAs green, sRNAs red, other RNAs black), GC content, GC skew. Replicons are not drawn to scale.

**Figure 4:**
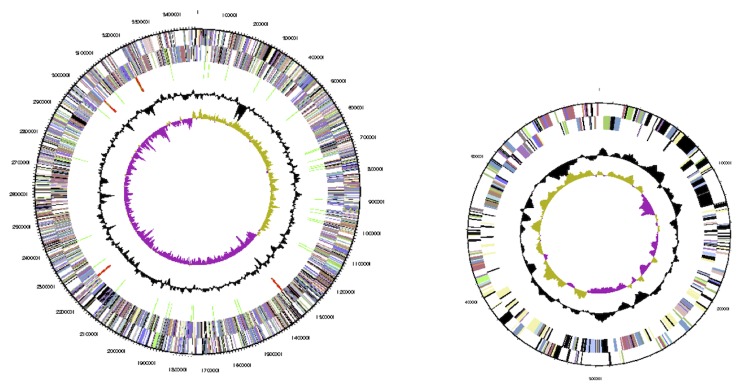
Circular maps of Plasmid 1 (left) and Plasmid 2 (right) of *B. phymatum* STM815^T^. From outside to center: Genes on forward strand (color by COG categories as denoted by the IMG platform), Genes on reverse strand (color by COG categories), RNA genes (tRNAs green, sRNAs red, other RNAs black), GC content, GC skew. Replicons are not drawn to scale.

The genome includes two chromosomes and two plasmids, for a total size of 8,676,562 bp (62.3% GC content). Chromosome 1 is 3.48 Mb in size (63.0% GC), chromosome 2 is 2.69 Mb (62.3% GC), plasmid 1 is 1.90 Mb (62.0% GC) and plasmid 2 0.59 Mb (59.2% GC). For chromosomes 1 and 2, 3,140 and 2,358 genes were predicted, respectively. For plasmid 1 and 2, 1,627 and 449 genes were predicted, respectively. A total 7,496 of protein coding genes were predicted, of which 5,601 were assigned to a putative function with the remaining annotated as hypothetical proteins. 5,630 protein coding genes belong to COG families in this genome. The properties and the statistics of the genome are summarized in Tables 4-6, and circular maps of each replicon are shown in Figure 3 (chromosomes) and Figure 4 (plasmids). Plasmid 2 was identified as the symbiotic plasmid of STM815, as it carried *nod*, *nif* and *fix* genes directly involved in symbiosis as well as several other genes coding for proteins indirectly linked to symbiotic interactions with plants. Among them were found genes coding for the biosynthesis of phytohormones such as indol acetic acid (*iaaHM*), ACC deaminase (*acdS*), and genes involved in the biosynthesis of rhizobitoxine (*rtxAC*-like). A Type 4 secretion system was also identified on this plasmid, while no type 3 system could be detected in the whole genome.

**Table 4:** Summary of genome: two chromosomes and two plasmids

**Label**	**Size (Mb)**	**Topology**	**INSDC identifier**	**Refseq identifier**
Chromosome 1	3.479189	Circular	NC_010622.1	CP001043.1
Chromosome 2	2.697376	Circular	NC_010627.1	CP001044.1
Plasmid 1	1.904895	Circular	NC_010623.1	CP001045.1
Plasmid 2	0.595110	Circular	NC_010625.1	CP001046.1

**Table 6:** Number of genes associated with the 25 general COG functional categories

**Code**	**Value**	**%age**^a^	**Description**
J	195	3.02	Translation
A	1	0.02	RNA processing and modification
K	643	10.00	Transcription
L	235	3.65	Replication, recombination and repair
B	2	0.03	Chromatin structure and dynamics
D	37	0.58	Cell cycle control, mitosis and meiosis
V	68	1.06	Defense mechanisms
T	397	6.17	Signal transduction mechanisms
M	396	6.16	Cell wall/membrane biogenesis
N	113	1.76	Cell motility
W	1	0.02	Extracellular structures
U	139	2.16	Intracellular trafficking and secretion
O	213	3.31	Posttranslational modification, protein turnover, chaperones
C	503	7.82	Energy production and conversion
G	486	7.55	Carbohydrate transport and metabolism
E	663	10.31	Amino acid transport and metabolism
F	101	1.57	Nucleotide transport and metabolism
H	223	3.47	Coenzyme transport and metabolism
I	290	4.51	Lipid transport and metabolism
P	287	4.46	Inorganic ion transport and metabolism
Q	200	3.11	Secondary metabolites biosynthesis, transport and catabolism
R	719	11.18	General function prediction only
S	522	8.11	Function unknown
-	1944	25.67	Not in COGs

a) The total is based on the total number of protein coding genes in the annotated genome.

**Table 5:** Nucleotide content and gene count levels of the genome

**Attribute**	Value	% of total^a^
Genome size (bp)	8676562	100.00%
DNA coding region (bp)	7328930	84.47%
DNA G+C content (bp)	5404839	62.29%
Total genes^b^	7574	100.00%
RNA genes	78	1.03%
Protein-coding genes	7496	98.93%
Genes assigned to COGs	5630	74.33%
Genes with signal peptides	701	9.26%
Genes with transmembrane helices	1709	22.56%

a) The total is based on either the size of the genome in base pairs or the total number of protein coding genes in the annotated genome.

b) Also includes 39 pseudogenes.

## Comparison of *Burkholderia phymatum* STM815^T^ with other fully sequenced genomes of *Burkholderia*

### Venn diagram (family number)

**Figure 5:**
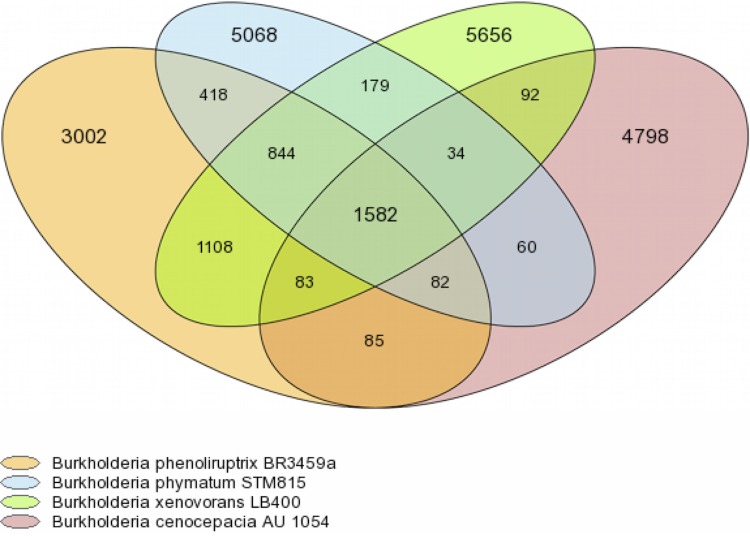
*B. phymatum* STM815^T^ was compared to 3 others *Burkholderia* strains from similar and different ecological niches: a legume symbiont (*B. phenoliruptrix* BR3459a, a *Mimosa flocculosa* nodule symbiont from Brazil [37,38]; a soil bacterium (*B. xenovorans* LB400) and a human opportunistic pathogen (*B. cenocepacia* AU1054). The core genomes of all four bacteria yielded 1,582 gene families. Each bacterium had more gene families specific to its species, (from 3,002 to 5,656 depending on strain) than shared ones (1,582 core gene families). There were 418 gene families specific to the two *Mimosa* symbionts (STM815 and BR3459a), including symbiosis-related genes (*nod* genes) and nitrogen fixation genes (*nif*, *fix*), glutamine transporters, biosynthesis genes of the phytohormone indol acetic acid (IAA), and hydrogenase genes (*hup*, *hyp*).

Gene families specific to, or shared by, *Burkholderia phymatum* STM815^T^ and 3 other *Burkholderia* species, were determined using MICFAM [Figure 5]. This tool is based on MicroScope gene families [39] which are computed using an algorithm implemented in the SiLiX software [40]: a single linkage clustering algorithm of homologous genes sharing an amino-acid alignment coverage and identity above a defined threshold. This algorithm operates on the “The friends of my friends are my friends” principle of gene comparison. If two genes are homologous, they are clustered. Moreover, if one of the genes is already clustered with another one, the three genes are clustered into the same MICFAM.

## Conclusion

*Burkholderia phymatum* STM815^T^ possesses a large genome composed of two chromosomes and two plasmids, one of which encodes the symbiotic functions. Further studies on the genome of this bacterium will help elucidate the high nodulation competitiveness [41], broad host range and symbiotic efficiency of this strain.
